# Rapid infusion rituximab is well tolerated in patients with primary CNS lymphoma

**DOI:** 10.2217/cns-2018-0001

**Published:** 2018-09-17

**Authors:** Lisa Modelevsky, Richard Tizon, Samantha N Reiss, Marcel Smith, Rachel Garonce, Thomas Kaley

**Affiliations:** 1 Memorial Sloan Kettering Cancer Center, 1275 York Ave, New York, NY 10065, USA

**Keywords:** neuro-oncology, primary CNS lymphoma, rapid infusion, rituximab

## Abstract

**Aim:**

To establish the safety and feasibility of rapidly infusing rituximab over 90 min in patients with primary CNS lymphoma (PCNSL).

**Patients & methods:**

We retrospectively reviewed all patients with PCNSL who received rapid rituximab infusions (RRI) from January 2016 to January 2017. Primary end point was incidence of infusion reactions.

**Results & conclusion:**

11 patients received a total of 44 RRIs. Rituximab was dosed at 500 or 750 mg/m^2^. Premedication included acetaminophen and diphenhydramine. No infusion reactions occurred during any RRI. Two infusions were administered with steroids for neurologic symptoms at baseline (4.5%). Rapid administration of rituximab was safe and feasible for patients with PCNSL and at the higher doses received.

Rituximab is a chimeric monoclonal antibody targeted against the CD20 antigen on B-lymphocytes. It is currently US FDA approved for treating non-Hodgkin's lymphoma (NHL), chronic lymphocytic leukemia (CLL), rheumatoid arthritis, granulomatosis with polyangiitis, and microscopic polyangiitis [1]. The addition of rituximab to standard chemotherapy has been shown to significantly improve overall survival for patients with systemic diffuse large B-cell lymphoma (DLBCL) [2–4]. In light of this data, rituximab was studied in combination with standard chemotherapy for primary CNS lymphoma (PCNSL) given that the histologic subtype in more than 90% of cases is DLBCL [5]. Based on the encouraging results from several studies, rituximab has been incorporated into the majority of standard induction regimens for CD20-positive PCNSL [6–16]. Today, upfront treatment of PCNSL primarily consists of induction chemoimmunotherapy with rituximab and methotrexate-based regimens (most commonly methotrexate, cytarabine, thiotepa, rituximab [MATRix], or rituximab, methotrexate, vincristine, procarbazine [RMVP], or methotrexate, temozolomide, rituximab [MTR]) [7,8,13,14,17,18]. In these regimens, rituximab is dosed between 375 and 500 mg/m^2^. Induction therapy is then generally followed by consolidation with high-dose chemotherapy, whole-brain radiotherapy or high-dose chemotherapy in combination with autologous stem cell transplant [7,18]. Rituximab has also been studied in the maintenance setting for patients with PCNSL unable to tolerate more aggressive consolidation. In the study conducted by Ney *et al*., maintenance rituximab was dosed at 750 mg/m^2^ and administered every 1–3 months [19].

Rituximab is generally well tolerated, though infusion-related reactions (IRRs) are common. At standard infusion rates, grade 3 and 4 IRRs occur in approximately 7% of NHL and CLL patients during the first infusion, 2% during fourth infusion, and 0% during the eighth infusion [1]. These reactions may range from fever, chills, rigors and hypotension to more severe reactions including pulmonary events, urticaria, anaphylaxis or death [1]. To minimize the risk of IRRs, the FDA-approved label recommends premedicating patients with acetaminophen and an antihistamine. The first infusion is initiated at a rate of 50 mg/h. In the absence of an IRR, the infusion rate is then increased by increments of 50 mg/h every 30 min to a maximum of 400 mg/h. Subsequent infusions are initiated at 100 mg/h and in the absence of IRR increased by 100 mg/h every 30 min to the same maximum rate. This labor-intensive process averages 4–5 h for the first infusion and 3 h for subsequent infusions. To reduce patient inconvenience and the burden on healthcare resources, a 90-min infusion schedule was previously developed and analyzed for safety and tolerability in patients with systemic lymphoma [20]. The results of a Phase III rapid infusion study led to the FDA's approval of a 90-min infusion in 2012. This study was conducted in 363 patients with previously untreated systemic NHL receiving rituximab dosed at 375 mg/m^2^ as part of standard treatment with rituximab, cyclophosphamide, doxorubicin, vincristine, and prednisone or rituximab, cyclophosphamide, vincristine, and prednisone. Patients who did not experience a grade 3 or 4 IRR during cycle 1 were eligible for a 90-min infusion during cycles 2–8. The incidence of grade 3 or 4 IRRs during cycles 2–8 was 2.8% [21]. Comparable rates of IRRs have been reported in studies administering rituximab at both standard infusion rates and rapid infusion rates [20,22–24]. However, RRI studies thus far have not included patients with CNS lymphoma or the higher doses these patients may receive for improved brain penetration ranging from 500 to 750 mg/m^2^.

Patients with systemic B-cell mediated diseases being treated at our institution have been receiving RRI for several years. Though we did not foresee any clinical issues with administering RRIs to patients with PCNSL, these patients were initially excluded from this method of administration given the lack of safety data in this population and the higher doses they receive. To standardize administration, and to improve both resource utilization and patient satisfaction, our chemotherapy committee recently approved the expansion of RRI to patients with PCNSL. We sought to identify all patients with PCNSL who had received RRI and confirm if rapid infusion of rituximab over 90 min was both safe and feasible in these patients and at the higher doses they may receive.

## Methods

### Study design

This study was an Institutional Review Board-approved, single-center, retrospective chart review assessing patients with PCNSL who received RRI from the time of implementation in this population in January 2016 and until 1 year later in January 2017. The primary end point of this study was incidence of infusion reactions of any grade during RRI.

### Rapid rituximab infusion practice for PCNSL

Eligibility criteria for patients with PCNSL to receive RRI included age greater than 18 years, previous standard rate rituximab infusion(s) without infusion reaction of any grade and patients were not receiving chemotherapy on the same day. Concomitant corticosteroids were permitted in order to control disease-related neurologic symptoms. If chemotherapy was part of treatment, rituximab was administered the day prior.

Patients who met criteria for RRI received the first 50 ml of the infusion (∼20% of total volume) over 30 m and the remaining volume over 1 h. Premedication was administered 30 min prior to infusion and consisted of acetaminophen 650 mg orally and diphenhydramine 25 mg intravenously. Rituximab was diluted in normal saline to a concentration of ≤ 4 mg/ml. Patients were monitored during RRI for signs of infusion reaction and vitals were measured after completion of infusion. If an IRR occurred, the infusion would be held until symptoms improved and then resumed at one-half of the rate at which reaction occurred. In the absence of IRR, patients continued to receive RRI for their remaining cycles.

### Data analysis

Patient information was reviewed from pharmacy databases and medical records. IRRs were characterized according to the Common Terminology Criteria for Adverse Events version 4.03 [25]. Descriptive statistics were implemented to describe patient characteristics and incidence of IRRs.

## Results

From January 2016 to January 2017, 17 patients with PCNSL were screened for RRI eligibility; six were excluded due to previous IRR. 11 patients (65%) were eligible for RRI and received a total of 44 RRIs over the 1 year period. The majority of patients had a histologic subtype of DLBCL (91%) and two patients (18%) had leptomeningeal disease. All patients had a normal absolute lymphocyte count, and median Karnofsky Performance Status was 90. Lactate dehydrogenase (LDH) was available for seven patients and was elevated in three of these patients prior to the first RRI. Baseline characteristics for patients who received RRI are provided in Table 1.

**Table 1.** **Baseline patient characteristics.**

**Patient characteristics**	**Subcharacteristics**	**n**	**(%)**
Number of patients		11	–

Median age (years; range)		61 (42–73)	–

Sex	Female	7	64

	Male	4	36

Race	Caucasian	10	91

	Asian	1	9

Median KPS (range)		90 (50–90)	–

Pathology	Diffuse large B-cell lymphoma	10	91

	Abnormal B-cell NOS (CD20+)	1	9

Presence of LMD		2	18

Maximum ALC (10^9^/l)	<1	3	27

	1–4	7	63

	>4	1	9

LDH (U/l)	Unavailable	4	36

	≤250	4	36

	>250	3	27

Chemotherapy regimen	R-MVP	7	64

	R-MBVP	1	9

	Maintenance every 2 months	3	27

ALC: Absolute lymphocyte count; KPS, Karnofsky performance status; LDH: Lactate dehydrogenase; LMD: Leptomeningeal disease; NOS: Not otherwise specified; R-MBVP: Rituximab, methotrexate, carmustine, etoposide, prednisone; R-MVP: Rituximab, methotrexate, vincristine, procarbazine.

Patients received RRI either in conjunction with chemotherapy (73%) or as single-agent maintenance therapy (27%). The majority of patients received rituximab as part of RMVP which includes rituximab dosed at 500 mg/m^2^ on day 1, methotrexate 3.5 g/m^2^ and vincristine 1.4 mg/m^2^ on day 2, all administered every 14 days for up to 8 cycles with the addition of procarbazine 100 mg/m^2^/d on days 1 through 7 of odd cycles (64%). Ten patients received rituximab dosed at 500 mg/m^2^ and one patient received 750 mg/m^2^; doses were not capped. The median number of RRIs per patient was four (range 1–7) since most patients had already received more than one of their planned rituximab infusions at standard rate prior to expansion of RRI to PCNSL patients. The median dosage received was 950 mg (range, 750–1500 mg). Characteristics of RRIs are provided in Table 2.

**Table 2.** **Rapid rituximab infusion characteristics.**

**Characteristics**	**n**	**(%)**
Total number of rapid infusions	44	100

Median number of infusions per patient (range)	4	(1–7)

Number of infusion reactions during rapid	0	0

Median rituximab dose in milligrams (range)	950	(750–1500)

Patients receiving 1 standard infusion prior to rapid	7	64

Patients receiving ≥2 standard infusions prior to rapid	4	36

Number of rapid infusions with concurrent steroids	2	5

All patients received standard premedication with acetaminophen and diphenhydramine. Two patients had been receiving dexamethasone 2 mg daily during the first RRI for disease-related neurologic symptoms; 42 of 44 RRIs (95%) were administered without steroids. All patients continued receiving RRI for entire duration of rituximab therapy.

Prior to the first RRI, seven out of 11 (64%) patients had received one standard infusion, one out of 11 (9%) had received three, and three out of the 11 (27%) patients had been receiving single-agent maintenance rituximab every 2 months at standard infusion rate for more than 2 years. No infusion reactions of any grade were observed during the 44 RRIs.

## Discussion

Rapid rituximab infusions were safe and feasible for all patients in this cohort. Patients did not experience IRRs whether they received RRI as part of chemoimmunotherapy or as single-agent maintenance therapy every 2 months. All but two infusions were administered without concomitant corticosteroids. Previous studies have also shown that RRIs are tolerated by patients whether administered with or without steroids [20,26].

Our practice differed from others in that only patients without an IRR during standard rate rituximab infusion(s) met criteria for RRI. This is different from the prescribing information which states that patients who experience a grade 1 or 2 reaction during cycle 1 may receive 90-min infusions starting with cycle 2. Our rationale for not administering RRI subsequent to an IRR was based on the lack of safety data for using RRI in this population and within this dose range. It is likely, however, that patients with PCNSL who experience grade 1 or 2 reactions during cycle 1 would tolerate RRIs with a low incidence of IRRs especially since these patients do not have systemic disease and do not have elevated absolute lymphocyte counts; a known predictor for IRR [27]. Our study also differed in that LDH was not available for all patients. We do not see this as a limitation since LDH is not routinely monitored for patients with PCNSL given the absence of systemic disease. Additionally, the doses of rituximab our patients received ranging from 500–750 mg/m^2^ were higher than those included in previous rapid rituximab studies for systemic NHL. Furthermore, the 750 mg/m^2^ dose is higher than FDA approved doses. It is important to note that these higher doses of rituximab used for PCNSL are considerably costlier than the standard doses used for systemic lymphoma, however, these higher doses were previously incorporated into studies primarily based on CSF pharmacokinetics. For patients with PCNSL, the efficacy of rituximab dosed at 375 mg/m^2^ has not been compared directly with 500 mg/m^2^ for induction. Lower dosages would afford cost savings although it is unknown if reducing the dosage would decrease efficacy.

The small sample size of our study is a limitation. However, given the rarity of PCNSL with an overall incidence rate of 0.45 per 100,000 persons, this study provides much-needed data to support the expansion of RRI to PCNSL patients receiving rituximab in the outpatient setting [28]. Based on the results of this study, patients with PCNSL who tolerate their first rituximab infusion administered at the standard rate may receive RRI starting with their second infusion. Patients who previously received more than one standard rituximab infusion and tolerated all prior infusions at standard rate may also be offered RRI.

Rapidly infusing rituximab over 90 min has been shown to save an average of 10.2 h per patient over the course of rituximab treatment, thereby improving patient satisfaction and offering an economic advantage by reducing chair time, resource utilization, and nursing monitoring. Additionally, patients with CNS disease were excluded from subcutaneous rituximab studies and it is unknown if this route of administration affects CNS penetration [29,30]. Therefore, this study provides essential safety and feasibility data supporting the expansion of RRIs to patients with PCNSL in an effort to decrease financial impact and improve patient satisfaction.

## Future perspective

Rituximab has become a standard component of treatment regimens for PCNSL. Most recently, the IELSG32 trial showed that the addition of rituximab to high-dose methotrexate and thiotepa improved overall response rates from 53 to 73% and improved the overall 2-year progression free survival from 36 to 46% [8]. Undoubtedly, rituximab will continue to be included in standard induction regimens unless in the years to come other treatments prove superior.  CSF levels of rituximab after intravenous administration are less than 1% of corresponding serum levels. As intravenous dosage increases, CSF concentration increases though it is unknown if higher concentration improves clinical outcomes [31]. Comparing the efficacy of lower versus higher doses of rituximab for the treatment of PCNSL may have further cost saving implications. An additional area of research that could further reduce healthcare costs and potentially improve patient satisfaction would be to determine if the efficacy of subcutaneously administered rituximab is comparable with intravenous administration for the treatment of PCNSL. Subcutaneous administration of rituximab results in lower peak serum concentrations versus intravenous administration [32,33]. Therefore, it is possible that subcutaneous administration may impair the penetration of rituximab across the blood–brain barrier; research is warranted before expanding this method of administration to all patients with PCNSL.

**Summary points**

- The safety and feasibility of rapidly infusing rituximab over 90 min has previously been established in patients with systemic B-cell lymphomas.
- Patients with CNS lymphoma were excluded from rapid infusion studies.
- Given the lack of data with rapid infusion in patients with CNS lymphoma, it was previously unknown if this faster rate of administration was also safe and feasible in this population.
- We retrospectively reviewed all patients with primary CNS lymphoma (PCNSL) who received rapid rituximab infusion over 90 min within a 1 year period.
- A total of 44 rapid infusions at doses of 500–750 mg/m^2^ were administered to this population.
- Patients received standard premedication with acetaminophen and diphenhydramine.
- Only two infusions were administered with steroids which were indicated for neurologic symptoms at baseline.
- No infusion reactions of any grade were experienced by patients with PCNSL.
- This study supports the expansion of rapid infusion rituximab to patients with PCNSL and at the higher dosages received affording an economic advantage over standard infusion times.
- Subcutaneous administration cannot currently be recommended for this population due to the lack of efficacy data in patients with PCNSL.
